# Evaluation of PET/MRI for Tumor Volume Delineation for Head and Neck Cancer

**DOI:** 10.3389/fonc.2017.00008

**Published:** 2017-01-23

**Authors:** Kyle Wang, Brandon T. Mullins, Aaron D. Falchook, Jun Lian, Kelei He, Dinggang Shen, Michael Dance, Weili Lin, Tiffany M. Sills, Shiva K. Das, Benjamin Y. Huang, Bhishamjit S. Chera

**Affiliations:** ^1^Department of Radiation Oncology, University of North Carolina Hospitals, Chapel Hill, NC, USA; ^2^State Key Laboratory for Novel Software Technology, Nanjing University, Nanjing, China; ^3^Department of Radiology, University of North Carolina Hospitals, Chapel Hill, NC, USA

**Keywords:** PET/MRI, CT, GTV, radiation treatment planning, head and neck cancer

## Abstract

**Introduction:**

Computed tomography (CT), combined positron emitted tomography and CT (PET/CT), and magnetic resonance imaging (MRI) are commonly used in head and neck radiation planning. Hybrid PET/MRI has garnered attention for potential added value in cancer staging and treatment planning. Herein, we compare PET/MRI vs. planning CT for head and neck cancer gross tumor volume (GTV) delineation.

**Material and methods:**

We prospectively enrolled patients with head and neck cancer treated with definitive chemoradiation to 60–70 Gy using IMRT. We performed pretreatment contrast-enhanced planning CT and gadolinium-enhanced PET/MRI. Primary and nodal volumes were delineated on planning CT (GTV-CT) prospectively before treatment and PET/MRI (GTV-PET/MRI) retrospectively after treatment. GTV-PET/MRI was compared to GTV-CT using separate rigid registrations for each tumor volume. The Dice similarity coefficient (DSC) metric evaluating spatial overlap and modified Hausdorff distance (mHD) evaluating mean orthogonal distance difference were calculated. Minimum dose to 95% of GTVs (D95) was compared.

**Results:**

Eleven patients were evaluable (10 oropharynx, 1 larynx). Nine patients had evaluable primary tumor GTVs and seven patients had evaluable nodal GTVs. Mean primary GTV-CT and GTV-PET/MRI size were 13.2 and 14.3 cc, with mean intersection 8.7 cc, DSC 0.63, and mHD 1.6 mm. D95 was 65.3 Gy for primary GTV-CT vs. 65.2 Gy for primary GTV-PET/MRI. Mean nodal GTV-CT and GTV-PET/MRI size were 19.0 and 23.0 cc, with mean intersection 14.4 cc, DSC 0.69, and mHD 2.3 mm. D95 was 62.3 Gy for both nodal GTV-CT and GTV-PET/MRI.

**Conclusion:**

In this series of patients with head and neck (primarily oropharynx) cancer, PET/MRI and CT-GTVs had similar volumes (though there were individual cases with larger differences) with overall small discrepancies in spatial overlap, small mean orthogonal distance differences, and similar radiation doses.

## Introduction

Head and neck radiation oncologists routinely utilize computed tomography (CT), combined positron emitted tomography and CT (PET/CT), and magnetic resonance imaging (MRI) for staging, treatment planning, and assessment of disease response. PET/CT has been shown to be superior to CT alone in staging head and neck cancer, with better sensitivity for nodal disease as well as distant metastases (1–3). However, MRI has potential advantages over CT, including detection of perineural spread, vascular involvement, and invasion of adjacent structures, as well as better soft tissue contrast (4–8). In addition, diffusion-weighted MRI imaging has been investigated as a method of increasing specificity for detection of nodal metastases and may add further benefit (9). The advantages of both PET/CT and MRI over CT alone have led to research into the role of hybrid PET/MRI imaging. This technology allows for simultaneous data acquisition resulting in optimal spatial and temporal co-registration of structural, functional, and molecular image data.

Combined positron emitted tomography and CT images are commonly utilized for delineation of the gross target volume (GTV) during radiotherapy treatment planning either with the use of dedicated PET/CT simulators or by registration to planning CT scan. However, advantages of MRI such as those discussed above could theoretically improve the accuracy of tumor delineation with PET/MRI vs. PET/CT. Furthermore, PET/CT images are acquired using sequential image registration, which may introduce spatial errors (though this may be mitigated with mask immobilization). In contrast, PET and MRI sequences are acquired simultaneously, which may reduce registration error (though ultimately this benefit may be limited by the need for a subsequent fusion to a treatment planning CT) (10). Nonetheless, the better soft tissue contrast of MRI combined with simultaneously acquired metabolic PET data may improve the accuracy and precision of GTV delineation, which could translate into changes in the clinical target volume (CTV) and/or affect the size of subsequent margins due to heightened confidence. These changes could have clinical implications for patients with regards to both disease control and treatment toxicity.

Previous studies have shown that use of PET/CT for treatment planning can sometimes alter GTV contours (11–13). Furthermore, PET/CT may more broadly impact treatment decisions, such as coverage of regional lymph nodes due to the increased sensitivity for nodal and distant disease. There have been several reports on the use of hybrid PET/MRI in the staging and posttreatment surveillance of head and neck cancer (14–19). However, there are no reports to our knowledge that have examined the usefulness of PET/MRI for radiation treatment planning for head and neck cancer. Delineating the primary tumor during head and neck radiation planning is challenging and relies on both physical exam and imaging findings, especially in the oropharynx, where borders between tumor and surrounding soft tissue may be indistinct. Therefore, we hypothesized that PET/MRI could improve contour accuracy in this setting. The purpose of this study is to assess differences in GTV delineation using hybrid PET/MRI vs. CT and to analyze CT-planned radiation dose coverage of the PET/MRI-delineated GTV.

## Materials and Methods

### Patient Population

We prospectively enrolled patients aged 18 years or higher with squamous cell carcinoma of the pharynx, larynx, or oral cavity who were treated definitively with radiation plus or minus concurrent chemotherapy with curative intent. Patients were excluded if they had an allergy to gadolinium, creatinine clearance <60 ml/min, were claustrophobic, or had an implanted pacemaker or other metallic devices. This study was carried out in accordance with the recommendations of the University of North Carolina Institutional Review Board with written informed consent from all subjects. All subjects gave written informed consent in accordance with the Declaration of Helsinki. The protocol was approved by the University of North Carolina Institutional Review Board (Lineberger Comprehensive Cancer Center 1020, IRB Study #10-1451).

### Pretreatment Imaging

Patients received treatment-planning CT simulation and PET/MRI scans 1–2 weeks prior to initiating radiotherapy. Treatment planning CT scans were obtained using a Philips Brilliance Big Bore Oncology CT scanner using intravenous iodinated contrast and with slice thickness ≤3 mm. Patients were immobilized using an aquaplast U-frame mask form fitted on the patient’s face and locked on to the table.

Positron emitted tomography/MRI scans were obtained after a 6-h fasting period. Patients received 10 mCi of intravenous 18F-FDG, 40–45 min prior to image acquisition. PET/MRI scans were conducted using a 3T Siemens Biograph mMR scanner. After acquiring localization images, MR attenuation images were acquired using the Dixon approach with a coronal 2-point 3D T1-weighted volumetric interpolated breath hold examination (VIBE) Dixon sequence [repetition time (TR) = 3.6 ms, echo time (TE) = 1.23 and 2.46 ms, 10° flip angle, 192 × 121 matrix, 500 mm × 328 mm field of view (FOV), 3.12 mm slice thickness, 20% interslice gap, one signal average, integrated parallel acquisition technique (iPAT) factor 2, acquisition time 19 s].

For the earlier patients recruited for the study, the anatomic MR imaging included pre- and postcontrast axial T1-weighted fast low angle shot (FLASH) images (TR = 310 ms, TE = 2.46 ms, 76° flip angle, 320 × 256 matrix, 230 mm × 230 mm FOV, 3 mm slice thickness with a 20% interslice gap, three signal averages, acquisition time 4 min) and fat-suppressed turbo spin echo T2-weighted images (TR = 8,260 ms, TE = 100 ms, 150° flip angle, 512 × 256 matrix, 230 mm × 230 mm FOV, 3 mm slice thickness with a 20% gap, two signal averages, iPAT factor 2, acquisition time 2 min 55 s). For patients enrolled later in the study, the T1 FLASH acquisitions were replaced with a fat-suppressed T1-weighted VIBE sequences (TR = 4.8 ms, TE = 2.18 ms, 9° flip angle, 256 × 256 matrix, 240 mm × 217 mm FOV, 0.9 mm slice thickness with a 20% gap, two signal averages, iPAT factor 2, acquisition time 6 min 55 s) Post-contrast images were obtained following intravenous injection of 0.2 mmol/kg of gadopentetate dimeglumine. Total “scan time” ranged from 30 to 45 min. PET/MRI scans were not obtained with immobilization in the treatment position.

### GTV-CT Delineation and Treatment

Contours for primary and nodal tumor volumes were drawn separately. Primary and nodal gross tumor volumes were delineated by the treating radiation oncologist on the contrast-enhanced treatment planning CT (GTV-CT) using the departmental treatment planning software Plan UNC (20) without using any other scans to aid in contouring. The primary GTV-CT was non-uniformly expanded using clinical judgment to form the high-risk primary CTV (for patients in this study, the median “minimum” primary CTV expansion was 7 mm, range 4–9 mm). The nodal GTV-CT was uniformly expanded by 7 mm to form the high-risk nodal CTV. An elective nodal region was contoured to form the standard risk CTV. All CTVs were expanded by 3 mm to form the corresponding planning target volume (PTV). All patients were treated using these CT-based volumes with intensity-modulated radiation therapy, using the Tomotherapy (R) Treatment Planning System (Accuray Inc., Sunnyvale, CA, USA) for nine patients and conventional linear accelerators for two patients (one on a Siemens Oncor and one on a Siemens Artiste, treatments planned using Plan UNC). Dosimetric calculations were performed using Tomotherapy-specific software for the patients treated on Tomotherapy, and Plan UNC for patients treated on conventional linear accelerators. Patients were treated using a simultaneous integrated boost technique to two prescribed dose levels: the high-risk and standard risk regions described above. Prescribed radiation dose to the high-risk volume was 60 or 70 Gy (2 Gy per fraction, seven patients were enrolled on an institutional de-intensification trial and received 60 Gy). Prescribed radiation dose to the standard risk volume was 54 Gy (1.8 Gy per fraction for patients prescribed 60 Gy to the high-risk volume, and 1.54 Gy per fraction for patients prescribed 70 Gy to the high-risk volume).

### GTV-PET/MRI Delineation

Following treatment of patients using CT-based volumes and plans, a *post hoc* analysis was performed using PET/MRI. PET/MRI scans were obtained pretreatment but were not used prospectively and did not influence radiation treatment planning. Primary and nodal volumes were delineated on PET/MRI (GTV-PET/MRI) using MIM Vista (MIM Software Inc., Cleveland, OH, USA). The GTV-PET/MRI was the union of a MRI-based and PET-based GTV. The MRI-based GTV was delineated manually using both contrast-enhanced T1 sequences and T2 sequences. The PET-based GTV was delineated using the MIM Vista PET Edge detection tool, which is a gradient-based method that has been previously described (21). The GTV-PET/MRI was delineated by the treating radiation oncologist and a single neuroradiologist without comparison to GTV-CT.

### Volumetric Evaluation

Volumetric evaluations were performed using MIM Vista and examined spatial and positional differences between GTV-CT and GTV-PET/MRI. Planning CT-based target contours and dose grids from each patient’s actual radiation treatment were exported to MIM Vista. PET/MRI scans were registered to the planning CT using the MIM Vista rigid registration tool per the technique as described by Hwang et al., where separate rigid registrations are manually performed for the primary as well as each nodal GTV “area of interest” using the closest anatomical landmarks for each instance. For our primarily oropharyngeal cancer patients, the landmarks most commonly used were the mandible, hyoid, vertebrae, enhancing blood vessels, and the tumor itself, if visible. In the Hwang study, this rigid registration process was found to have an error of roughly 4 mm for tumor volumes, as assessed using the COM (distance between calculated centers of mass for two volumes) method (22).

The volumes and intersection of both primary and nodal GTV-CT and GTV-PET/MRI contours were then recorded to calculate the Dice similarity coefficient (DSC), a validated metric to evaluate spatial overlap between two volumes. The DSC is calculated using the equation: 2 × (A ∩ B)/(A + B), where A and B represent two volumes, (A ∩ B) represents the volume of intersection, and (A + B) represents the absolute sum of their volumes. A DSC ≥0.7 has been reported as a “good” overlap by some investigators (23), but DSC may also vary with changes in the size of the compared volumes. In addition, the modified Hausdorff distance (mHD) (24) was calculated for each GTV-CT and corresponding GTV-PET/MRI contour. The mHD measures the similarity between two volumes by reporting the mean orthogonal distance between surface points. A smaller value suggests a lower “distance” and, therefore, greater similarity between the compared volumes.

### Dosimetric Evaluation

**Figure 1:**
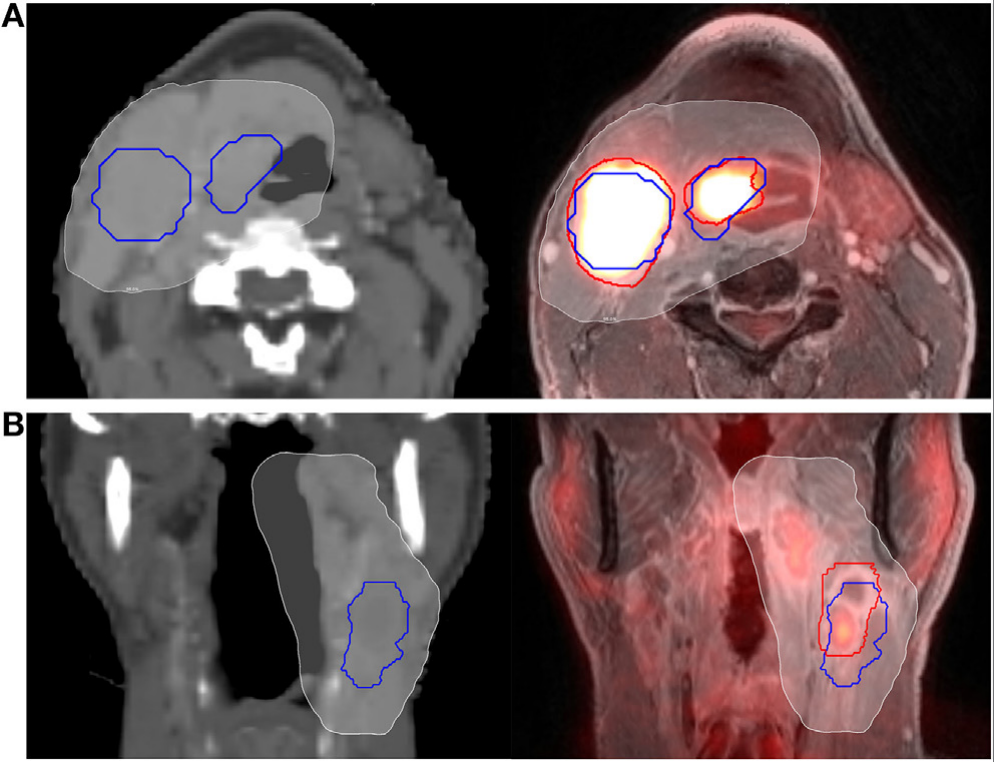
**Example of computed tomography (CT) vs. PET/MRI GTV delineation**. Primary tumor and nodal GTVs for two different patients **(A,B)** are shown delineated using CT (blue outline) and PET/MRI (red outline). The 95% prescription isodose line from their CT-based treatment plan is overlayed (white, shaded). The patient in panel **(A)** (axial representation) had fair spatial overlap between PET/MRI and CT volumes, with a Dice similarity coefficient (DSC) of 0.72 for the primary GTV and 0.69 for the nodal GTV. The patient in panel **(B)** (coronal representation) had relatively poor spatial overlap between the PET/MRI and CT nodal volumes, with a DSC of only 0.48. Clinical target volume and planning target volume expansions are not shown.

Dosimetric evaluation was performed using MIM Vista. As discussed above, GTV-CT was used in treatment planning, whereas GTV-PET/MRI was delineated for research purposes only. The purpose of the dosimetric evaluation was to assess the clinical significance of any volumetric differences; does a treatment plan created using GTVs delineated on CT only (as is standard at many institutions) potentially underdose GTVs as delineated on PET/MRI? For both primary and nodal tumor volumes, doses for the registered GTV-PET/MRI and GTV-CT were calculated, including the minimum dose delivered to the entire GTV (D100) and the minimum dose delivered to 95% of the GTV (D95), representing the “hottest” 95% of the GTV. Figure 1 depicts examples of GTV delineation, dose coverage, and assessment of spatial overlap between planning CT and PET/MRI for two patients.

### Statistical Considerations

Statistical analyses were performed using IBM SPSS Statistics for Windows, Version 21.0 (IBM Corp., Armonk, NY, USA). Non-parametric paired-sample Wilcoxon signed-rank test was used to compare GTV size and dose received by CT vs. PET/MRI-generated volumes, with a two-sided *p* value <0.05 considered statistically significant.

## Results

Patient characteristics are summarized in Table 1. There were 11 patients who received both a pretreatment planning CT and PET/MRI and were evaluable. PET/MRI was performed on the same day as planning CT for six patients and within 5 days of planning CT for the remaining five patients. No stage migration was observed. Ten patients had primary oropharyngeal carcinoma and one patient had laryngeal carcinoma. Ten patients received chemoradiation and one patient received radiation alone. Of the 11 enrolled patients, 9 patients had evaluable primary GTVs (2 patients had unknown primary) and 7 patients had evaluable nodal GTVs (4 patients had node-negative disease).

**Table 1:** **Patient characteristics (*n* = 11)**.

**Primary tumor site**	
Tonsil	7 (64%)
Base of tongue	3 (27%)
Larynx	1 (9%)
**T-stage**	
T1	2 (18%)
T2	7 (64%)
T3	1 (9%)
T4	1 (9%)
**N-stage**	
N0	3 (27%)
N1	1 (9%)
N2b	6 (55%)
N2c	1 (9%)
**Prescribed radiation dose**	
70 Gy	4 (36%)
60 Gy	7 (64%)
**Unilateral vs. bilateral neck radiation**	
Unilateral	3 (27%)
Bilateral	8 (73%)

**Figure 2:**
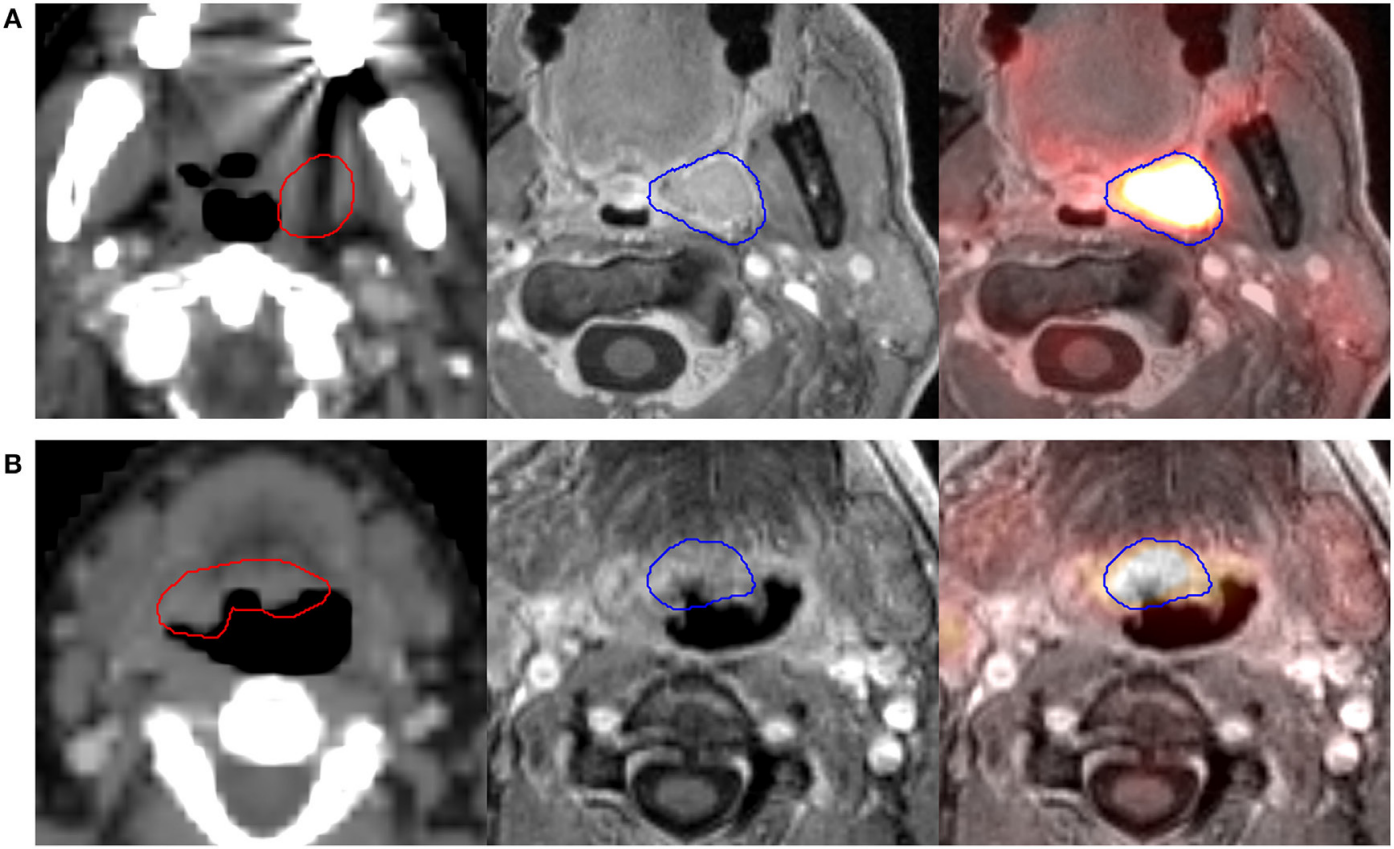
**Two different patients [(A), patient 7, and (B), patient 11] where PET/MRI substantially altered the primary GTV**. Scans from left to right are the planning computed tomography (CT) scan (GTV-CT shown in red), magnetic resonance imaging (MRI) component of PET/MRI, and fused PET/MRI (GTV-PET/MRI shown in blue). Patient 7 **(A)** had a left tonsil primary. PET/MRI showed soft tissue encroachment to the uvula not delineated on CT, with a resultant *increase* in GTV size. Patient 11 **(B)** had a base of tongue primary. Though the planning CT-GTV included indeterminate soft tissue in the right base of tongue, PET/MRI showed tumor limited to the central base of tongue, with a resultant *decrease* in GTV size.

Results of the volumetric assessment of PET/MRI vs. planning CT-generated GTVs are shown in Table 2. Overall, GTVs generated by both imaging modalities were similar in size, with mean primary GTV of 13.2 and 14.3 cc (*p* = 0.82) and mean nodal GTV of 19.0 and 23.0 cc (*p* = 0.94), for CT and PET/MRI, respectively. However, there were individual cases with larger volumetric differences due to the improved visualization of gross tumor with PET/MRI (e.g., patients 7 and 11, Figure 2). There was some spatial discrepancy between the locations of the CT and PET/MRI volumes, with mean intersection of only 8.7 cc for primary and 14.4 cc for nodal tumors. The mean DSC between PET/MRI and CT was 0.63 (SD 0.11) for primary tumor volumes and 0.69 (SD 0.10) for nodal tumor volumes. Results of the mHD calculations are shown in Table 3. The mean mHD was 1.6 mm (SD 0.7 mm) between PET/MRI and planning CT primary tumor volumes and 2.3 mm (SD 1.5 mm) between nodal tumor volumes.

**Table 2:** **Volumetric comparison of computed tomography vs. PET/MRI GTVs**.

	Primary tumor volume (cc)				Nodal tumor volume (cc)			
Pt.	GTV-CT	GTV-PET/MRI	Intersection	DSC	GTV-CT	GTV-PET/MRI	Intersection	DSC
1	23.6	22.1	12.2	0.53	–	–	–	–
2	–	–	–	–	19.1	20.4	15.0	0.76
3	–	–	–	–	12.0	17.4	9.9	0.67
4	3.9	7.3	3.1	0.55	10.3	8.6	4.5	0.48
5	15.0	14.1	10.9	0.75	–	–	–	–
6	22.1	28.1	17.5	0.70	25.7	23.9	18.5	0.75
7	19.6	28.8	15.7	0.65	4.6	4.3	3.1	0.70
8	6.4	6.3	4.6	0.72	50.2	77.1	44.1	0.69
9	7.8	8.3	5.0	0.62	–	–	–	–
10	10.8	9.5	6.9	0.68	–	–	–	–
11	10.0	4.1	2.8	0.40	11.5	9.4	5.7	0.55
Mean (SD)	13.2	14.3	8.7	0.63 (0.11)	19.0	23.0	14.4	0.69 (0.10)

*GTV, gross tumor volume; DSC, dice similarity coefficient*.

**Table 3:** **Modified Hausdorff Distances for computed tomography vs. PET/MRI GTVs**.

Pt.	Primary tumor (mm)	Nodal tumor (s) (mm)
1	1.3	–
2	–	0.8
3	–	1.4
4	2.0	2.7
5	1.6	–
6	1.2	2.1
7	2.6	5.3
8	1.1	2.5
9	1.1	–
10	1.0	–
11	2.7	1.5
Mean (SD)	1.6 (0.7)	2.3 (1.5)

Results of the dosimetric evaluation are shown in Table 4. The small overlap discrepancy and small mHD between GTV-CT and GTV-PET/MRI did not appear to lead to potential underdosing of the GTV-PET/MRI based on treatment plans generated using planning CT alone. For both primary and nodal tumors, D100 and D95 were close to prescription dose and not significantly different between CT vs. PET/MRI-generated GTVs for each patient and on average. The D95 was very similar between CT and PET/MRI-generated GTVs, regardless of whether they received a prescription RT dose of 60 or 70 Gy.

**Table 4:** **Dosimetric comparison of computed tomography vs. PET/MRI GTVs**.

Pt.	Prescribed dose (Gy)	Primary tumor dose (Gy)				Nodal tumor dose (Gy)			
		D100 GTV-CT	D100 GTV-PET/MRI	D95 GTV-CT	D95 GTV-PET/MRI	D100 GTV-CT	D100 GTV-PET/MRI	D95 GTV-CT	D95 GTV-PET/MRI
1	70	70.8	65.8	71.1	71.1	–	–	–	–
2	60	–	–	–	–	60.8	60.8	61.4	61.4
3	60	–	–	–	–	60.5	60.3	61.2	61.1
4	60	60.5	58.5	61.0	60.3	60.7	56.7	61.0	60.7
5	70	70.5	70.5	70.9	70.9	–	–	–	–
6	60	59.6	59.6	60.2	60.2	60.0	60.0	60.4	60.4
7	70	71.0	70.4	71.4	71.3	70.5	70.6	71.1	71.2
8	60	60.3	60.1	60.8	60.7	59.9	54.7	60.8	60.6
9	70	70.9	70.5	71.2	71.2	–	–	–	–
10	60	60.3	60.4	60.7	60.8	–	–	–	–
11	60	59.8	59.9	60.2	60.2	60.0	59.9	60.3	60.3
Mean	63.6	64.8	64.0	65.3	65.2	61.8	60.4	62.3	62.3

*GTV, gross tumor volume; D100, dose received by 100% of the GTV; D95, dose received by 95% of the GTV*.

## Discussion

We assessed volumetric differences in GTVs delineated using routine contrast-enhanced planning CT vs. gadolinium-enhanced PET/MRI, as well as differences in dose received by these volumes after treatment based on planning CT alone. Overall, the mean primary and nodal GTV size for all patients was not significantly different between CT and PET/MRI volumes, though there were individual cases with larger differences (Figure 2). The use of PET/MRI led to volumes that were sometimes somewhat spatially discordant from CT volumes, with an average DSC slightly below 0.7 for both primary and nodal volumes. Furthermore, mean orthogonal distance difference as calculated using the modified Hausdorff method was low between CT and PET/MRI volumes. Given these similar volumes, small discrepancies in spatial overlap, and small mean orthogonal distance differences, doses received by PET/MRI GTVs were also similar to doses received by CT-GTVs based on CT-only treatment plans.

Target delineation for radiotherapy planning is dependent on quality of radiologic studies, and there is interest in hybrid PET/MRI systems incorporating benefits of both PET and MRI. Previous studies that have similarly investigated the usefulness of PET/CT for target delineation have shown varied results, though most suggest that PET/CT delineation reduces GTV size compared to CT alone. In one study, Leclerc et al. showed that PET-based contours were smaller and resulted in significantly lower parotid and oral cavity doses (12). Guido et al. also showed smaller GTV size with PET/CT vs. CT planning (11). However, Paulino et al. showed that volumes were larger in 18% of patients using PET/CT delineation, which resulted in underdosing of PET/CT-GTVs based on the CT-GTV radiation plan (13). To the authors’ knowledge, there are no similar studies comparing PET/MRI vs. CT planning, which was the impetus for this pilot study. Here, we report that in a predominantly oropharyngeal cancer patient population, PET/MRI and CT-GTVs were volumetrically and dosimetrically (based on CT planning) similar.

Regardless of any observed changes to the GTV using PET delineation, a larger question is whether any difference (between tumor delineation using CT vs. PET or any other imaging modality) is clinically significant. In our dosimetric analysis, we attempted to answer this question by assessing whether CT-generated treatment plans would have underdosed gross tumor as delineated on PET/MRI, which would have potentially important implications. We found that dosimetric coverage was not substantially different between PET/MRI and CT-based volumes, regardless of the degree of volumetric agreement. Similarly, in a previous report from our institution on 91 patients with head and neck cancer, GTVs based on pretreatment PET/CT scans were generated and registered to their CT-based treatment plans. There was no difference in radiation dose to PET/CT vs. CT-GTVs, even in patients who later developed local recurrence (25). These findings are most likely a reflection of CTV and PTV expansions. Regardless of small changes in GTV size, CTV expansions in the range of 5–10 mm are routinely applied at both the primary and involved nodal sites. Furthermore, an even larger region of “standard risk” CTV that covers uninvolved at-risk neck nodal levels is prescribed doses in the range of 50–60 Gy, and additional PTV margins of 3–5 mm are routinely added to each CTV.

Given that the mHD was only around 2 mm between PET/MRI and CT-GTVs in our study, any volumetric differences are likely to be masked by these expansions in terms of actual dose received. In another study, Fortunati et al. also investigated spatial and positional differences between registered head and neck volumes delineated on CT vs. MRI. Though their study examined normal tissue structures rather than tumor volumes, they reported a mean mHD of 1.7 mm, similar to our findings (26). Fleckenstein et al. compared lung cancer tumor volumes delineated on diffusion-weighted MRI registered to tumor volumes delineated using PET/CT and report a similar “average” Hausdorff distance of 2.25 mm (27). Furthermore, Commandeur et al. investigated prostate volumes delineated on MRI vs. CT and report a Hausdorff distance of around 3 mm (28). Taken as a whole, these small Hausdorff distances suggest that the benefit of alternate imaging modalities for gross tumor delineation may be greater in situations where large CTV/PTV margins are not routinely used or are limited by critical organs.

Although we do not report a benefit of PET/MRI here, this was a pilot study of a new technology, and there are numerous exciting aspects (and disease settings) that warrant further investigation. For instance, we did not examine the impact of PET/MRI on subjective CTV expansions, which ranged from 4 to 9 mm around the high-risk GTV using planning CT. There were several patients in this study where the use of PET/MRI substantially altered the GTV, whether increasing or decreasing the volume (as demonstrated in Figure 2). It is possible that heightened confidence in gross tumor delineation with PET/MRI could lead to the use of smaller CTV margins, which could reduce toxicity while maintaining confidence in tumor coverage. Furthermore, future studies could also investigate whether PET/MRI could reduce inter-observer variation in tumor volume delineation, which could translate to improved “quality” for head and neck cancer treatment.

Future studies on the utility of PET/MRI for tumor delineation may also benefit from incorporation of more advanced imaging techniques such as functional MR sequences and novel radiotracers. For instance, studies are underway to investigate the combination of PET with diffusion-weighted imaging (DWI) and MR spectrosocopy for infiltrative brain gliomas (29). In head and neck cancer, one area of particular interest is the potential ability of functional MRI sequences such as DWI to differentiate benign from malignant lymph nodes and thus increase the specificity of PET/MRI as compared to PET/CT. In one study, Schouten et al. reported that DWI-MRI had a higher specificity for nodal disease (93%) as opposed to PET/CT (84%) (9). In contrast, Queiroz et al. assessed the predictive value of DWI as part of PET/MRI staging in 70 patients and were unable to detect a difference in DWI parameters between benign and malignant lesions (30). Though further research is needed, if PET/MRI improves the “positive predictive value” for nodal disease, this could have major implications on treatment (patient selection, tumor, and CTV delineation) and follow-up (selection of patients for salvage surgery vs. continued surveillance). Another interesting avenue of research is the incorporation of new PET tracers with hybrid PET/MRI. Navarria et al. used Carbon-11-labeled methionine (11C-MET) PET/MRI in 69 patients with high grade glioma. Whereas 50% of failures were outside the T1 post-contrast volume, all failures would have been encompassed within a “biologic tumor volume” generated by use of the 11C-MET uptake regions (31). Furthermore, the use of novel radiotracers in combination with functional MRI sequences may be useful to assess intra-treatment (e.g., aiding in adaptive radiation planning) and posttreatment response.

A major limitation of the present study is the rigid registration process, which is expected to introduce some degree of error in the assessment of differences between PET/MRI and CT contours. In addition, PET/MRI scans were not specified to be completed in the treatment position, which may increase the inherent error when performing image registrations. We originally intended to use deformable registration, but we found that the deformable registration performance of MIM Vista Software when registering two different image modalities (e.g., MR to CT as in this study) was suboptimal. Therefore, we opted to use multiple rigid body registration. As newer deformable registration technology becomes available, it may become more feasible to perform cross modality (e.g., MRI to CT) deformable registration for comparison of tumor volumes. Also, the necessity of CT for radiation dose calculations and, therefore, PET/MRI registration is a significant challenge in the implementation of this technology for radiotherapy planning, though MRI planning is an area of current research interest (in contrast to PET/CT simulators, which are already in routine use). Furthermore, the longer image acquisition time of MRI may also introduce motion artifact and increase delineation error, though this limitation applies to most studies using this modality. Another limitation is that we did not assess intra or inter-observer variation; the same radiation oncologist generated the final volumes on both CT and PET/MRI (with the aid of a neuroradiologist). The number of patients was also limited, and it is possible that with more patients, we could have found more individuals in which PET/MRI had a greater impact. Our primary objective was to assess differences in GTV delineation and dosimetric coverage of this GTV, and we did not examine the impact of PET/MRI on CTV expansions (e.g., does heightened confidence decrease subjective margins?). Finally, we did not examine the impact of functional imaging sequences, including DWI-MRI and dynamic contrast-enhanced MRI, which could provide additional benefit.

Patients in our study had uncomplicated head and neck cancers, and although there were individuals with larger differences, our analysis showed that PET/MRI and CT-generated GTVs were overall similar volumetrically and received similar radiation doses. Further studies in this and other settings are needed to determine the optimal use of PET/MRI for treatment planning. The usefulness of PET/MRI for tumor delineation may also be greater in situations where the tumor is more difficult to delineate using standard radiographic studies, including situations such as re-irradiation and locations such as base of skull, nasopharynx, and paranasal sinuses, where perineural and intracranial extension are more difficult to ascertain. PET/MRI target delineation may also be advantageous for radiosurgery, particle therapy, and in locations juxtaposed to critical normal tissue structures, where allowable margins are small and dose gradients are very sharp. These are clinical situations where even small changes in GTV delineation could affect dosimetric coverage and potentially clinical outcomes.

## Author Contributions

KW—data analysis and interpretation, manuscript writing, and manuscript approval. BM—collection of data, manuscript writing, and manuscript approval. AF—collection of data, data analysis and interpretation, manuscript writing, and manuscript approval. JL, KH, and DS—collection of data, manuscript writing, and manuscript approval. MD—data analysis and interpretation, manuscript writing, and manuscript approval. WL—study conception and design, manuscript writing, and manuscript approval. TS—collection of data, manuscript writing, and manuscript approval. SD and BH—data analysis and interpretation, manuscript writing, and manuscript approval. BC—study conception and design, manuscript writing, and manuscript approval.

## Conflict of Interest Statement

The authors declare that the research was conducted in the absence of any commercial or financial relationships that could be construed as a potential conflict of interest.
